# Effect of home-based pulmonary rehabilitation on functional capacity in people with idiopathic pulmonary fibrosis—a systematic review protocol

**DOI:** 10.1186/s13643-021-01853-9

**Published:** 2021-11-15

**Authors:** Revati Amin, K. Vaishali, G. Arun Maiya, Aswini Kumar Mohapatra, Uday Narayan Yadav, Shradha S. Parsekar

**Affiliations:** 1 Department of Physiotherapy, Manipal College of Health Professions, Manipal Academy of Higher Education (MAHE), Manipal, India; 2 Department of Respiratory Medicine, Kasturba Medical College, MAHE, Manipal, India; 3 National Centre for Epidemiology and Population Health, Research School of Population Health, The Australian National University, Canberra, Australia; 4 Public Health Evidence South Asia, Prasanna School of Public Health, MAHE, Manipal, India

**Keywords:** Functional capacity, Home-based pulmonary rehabilitation, Health-related quality of life, Idiopathic pulmonary fibrosis, Systematic review

## Abstract

**Background:**

Idiopathic pulmonary fibrosis (IPF) is one of the common types of interstitial lung disease having high prevalence and mortality worldwide. As a result of patient-centred hindering factors of adherence to centre-based pulmonary rehabilitation (PR), home-based PR is an alternate mode of rehabilitating individuals with IPF. This systematic review will evaluate the effectiveness of unsupervised home-based PR on functional capacity and health-related quality of life (HRQoL) in individuals with IPF.

**Methods:**

Clinically stable, high resolution computed tomography and physician diagnosed IPF participants having modified Medical Research Council score below 5 will be considered for the systematic review. Studies involving home-based PR as an intervention to treat individuals with IPF will be considered. Randomised controlled trials and quasi-randomised studies (with two groups followed up) are eligible to be included. Outcomes of our interest are functional capacity (6-min walk distance, shuttle walk test and incremental shuttle walk test) and secondary outcome measure would include assessment of quality of life and adverse effects of intervention. Electronic databases such as SCOPUS, Medline (PubMed and Web of Science), PEDRo and CINAHL will be searched using database specific terms. Additionally, forward and backward citations of included studies will be searched to identify potential records. Two review authors, independently, will conduct the screening, data extraction using a customised standard tool, and critical appraisal using Cochrane Risk of Bias 2 tool of included studies. If data permits, meta-analysis will be conducted. In case of substantial heterogeneity, we will do a narrative synthesis. Subgroup analysis will be undertaken based on various contextual and interventional factors.

**Discussion:**

This review will provide comprehensive evidence on the effectiveness of unsupervised home-based PR to physiotherapists, policy makers and researchers who are interested in IPF management. Findings from this review may guide the development and evaluation of more robust evidence based home-based PR that aimed to improve functional capacity among people with IPF.

**Systematic review registration:**

PROSPERO CRD42020213883.

**Supplementary Information:**

The online version contains supplementary material available at 10.1186/s13643-021-01853-9.

## Background

‘Idiopathic pulmonary fibrosis’ (IPF), one of the prime variants of ‘idiopathic interstitial pneumonia’, is a recurrent, progressive, irreversible and generally lethal lung disease of unknown origin [1]. Men and women are frequently affected, with poor median survival; and the survival varies between 2 and 5 years [1, 2]. The incidence of IPF varies across the globe and ranges between 0.2 and 93.7 per 100,000 population per year [2]. There is diversity in age standardised mortality rate of IPF between different countries (4.64 to 8.28 per 100,000 population); however, data were mostly available from few high-income countries [2]. Increasing trend in IPF has been reported over the years in many countries [2]. Cigarette smoking and exposure to metal and wood emissions were the most important environmental threats for developing IPF [1, 3, 4].

IPF individuals seek treatment for chronic and progressive cough and dyspnoea. Dyspnoea and fatigue deteriorate functional ability and quality of life in individuals with IPF. As fibrosis progresses, dyspnoea and fatigue intensify, individual with IPF become gradually less physically involved and unable to perform physical tasks [5–7].

Pharmacotherapy (pirfenidone and nintedanib) is a promising approach in the management of mild to moderately impaired lung function tests among IPF [8]. Additionally, IPF involves the application of home-based pulmonary rehabilitation (PR) programme to optimise functional outcomes [9, 10]. Individually tailored exercise training is the cornerstone of PR. The exercise training component included both aerobic and strength training [9, 11] and each session may consist of up to 30 min of aerobic training [12]. PR, a systematic technique, improves dyspnoea, enhances exercise efficiency and improves health-related quality of life (HRQoL) [13]. PR is commonly delivered in an outpatient or community setting and may comprise of two - three sessions per week [14]. Disease-specific education and self-management training helps people with IPF to develop and implement the skills necessary to perform the tasks, guide lifestyle behaviour change and provide support to achieve optimal function [14, 15].

Despite the proven benefits of PR for individuals with ‘chronic respiratory diseases’ (CRD), a small percentage of individuals who are eligible to attend PR adhere to it on regular basis [16]. Some of the patient-centred barriers to attendance and adherence of PR are related to travel and transport to the rehabilitation centre. Lack of services and suitably trained healthcare professionals especially in non- metropolitan areas makes it difficult to avail the services [16, 17]. Home-based PR has the potential to overcome known barriers to PR participation and could be a relevant treatment alternative across all CRD including IPF [14].

The empirical research has shown the benefits of PR in enhancing functional capability and HRQoL, thereby minimising hospitalisation and frequency of hospital stay [18]. PR programmes are demonstrated to be successful by home-based mode of rehabilitation for training, and therefore, previous research had recommended creation of such programmes [19–21]. Home-based PR programmes, for a larger proportion of IPF individuals living in lesser developed countries or rural and remote surroundings, may contribute to broader provision of PR. Furthermore, unsupervised home-based PR can be a promising approach for a resource-limited remote area of the world.

Previous systematic reviews for supervised exercise training programmes have exhibited clinical benefits in ameliorating exercise capacity, dyspnoea and quality of life in individuals with IPF [9, 12, 22]. A systematic review [9] included studies conducted a mixture of centre- and home-based setting to assess the effects of exercise-based PR in individuals with IPF. As the subgroup analysis was not conducted, it is difficult to comment on which of the two settings proved to be beneficial. The underlying effects of chronic adaptation to a regular non-supervised home-based PR on functional capacity in IPF have yet to be described by a systematic review level evidence. There has not been a comprehensive assessment of the capacity of non-supervised home-based PR to achieve improvements in functional capacity using 6-min walk test [23], shuttle walk test [24], incremental shuttle walk test and HRQoL [18, 25, 26] in people with IPF or its ability to improve uptake and access to rehabilitation services.

The current systematic review is, therefore, planned to evaluate the effectiveness of an unsupervised self-care home-based PR training programme on the functional capacity in individuals with IPF. The evidence generated from this systematic review will guide policy makers, researchers and public health practitioners in designing evidence-based PR programme performed at home, which might help in improving the HRQoL, activity of daily living and bring about functional improvements among people with IPF. It would provide basis for establishing a standard protocol and an alternative to supervised centre-based rehabilitation programmes for individuals with IPF.

## Material and methods

This systematic review protocol has adhered to PRISMA-P guidelines [27]. The protocol is registered under PROSPERO, CRD42020213883.

### Inclusion and exclusion criteria

#### Participants

Individuals with IPF diagnosed using high-resolution computed tomography findings and by registered physician will be included. We have considered these inclusion criteria as per the definition provided by the American Thoracic Society/European Respiratory Society guidelines [1]. IPF could be of any severity, but the individual should be in a stable clinical state. Individuals with IPF having dyspnoea with modified Medical Research Council score of grade 5 would be excluded from the review. Studies on surgically treated lung transplanted IPF individuals will be excluded as their care and rehabilitation pathways differ significantly from those with IPF individuals without lung transplant. Studies with mixed population will be excluded; however, we will include the study if it has carried out subgroup analysis of population of our interest. We will exclude other forms of interstitial lung disease other than IPF. There would be no restriction on the duration of diagnosis of IPF and the age of the population.

#### Intervention

The intervention eligible to be included in this review is a comprehensive home-based PR, based on exercise training. Home-based PR is a self-care-based intervention, in which the physiotherapist/professional provides training and the participant perform the PR by themselves at home. This intervention is mostly unsupervised, but caregiver-supervised PR are eligible to be included. We will include studies with physiotherapist/any professionally trained individual periodically (minimum once in 15 days) supervising the participant, either at the participant’s home, community or hospital setup. This supervision could be web- or tele-based. Home-based PR may be performed in a group or individually in the community. To be included, studies must consider home-based PR programmes that have a component of aerobic exercise, resistance exercise, or both, with or without health education. Nevertheless, education programmes without home-based PR will be excluded. Minimum intervention duration should be 4 weeks but could be of any frequency per week. We will exclude studies that provided single exercise programmes. If the first and single training PR session has been delivered at centre/hospital, but remaining sessions were carried out at home/community setup, we will include the study. We will consider studies with individuals undergoing pharmacotherapy or any other standard care, but the co-intervention should have been equally distributed in both the groups. If the study included mixture of home-based and centre-based PR, we would include it provided there is subgroup available for unsupervised home-based PR.

#### Comparisons

Conventional supervised, centre-based PR treatment, no treatment or standard care is included in comparisons. We will include studies that compare unsupervised home-based PR with traditional/conventional PR or no rehabilitation. Comparison could be drawn with centre-based PR or between the providers/supervisors. Within home-based PR, comparison could be between two different forms (e.g. aerobic home-based PR compared to strength training home-based PR compared to strength training home-based PR), duration or intensity.

#### Outcomes

Primary outcome measures are functional capacity, as measured by 6-min walk distance, shuttle walk test or incremental shuttle walk test. Secondary outcome measures are condition specific quality of life measured using scales such as St. George respiratory questionnaire [26], Chronic Respiratory Distress Questionnaire [17] and King’s Brief Interstitial Lung Disease Questionnaire [28, 29]. Studies assessing outcome measures at time period of at least 4 weeks post intervention will be considered. We will exclude other generic tools for assessing quality of life such as WHO BREF and SF-36 (Short form- 36). We will also measure side-effects of PR such as fatigue and muscle weakness but not limited to desaturation and exacerbation of dyspnoea.

#### Study type

Randomised controlled trials (RCTs) with a parallel, cluster or cross-over design and quasi-randomised studies with at least two groups (intervention and control), followed-up for at least 4-week duration, will be included. Non-randomised studies (with single group), observational studies, letters to editor and reviews will be excluded.

### Conceptual framework of the current systematic review

**Fig. 1:**
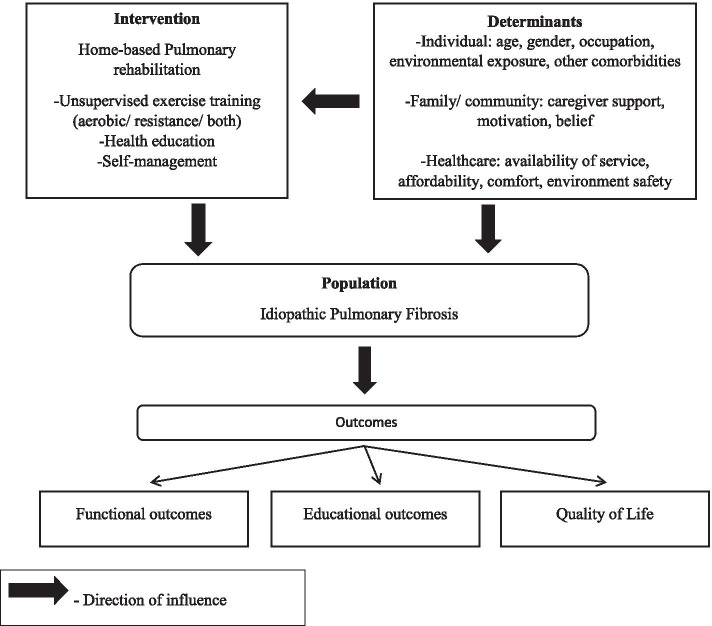
Conceptual framework for the effects of home-based pulmonary rehabilitation on individuals with idiopathic pulmonary fibrosis (authors creation of the figure)

We hypothesise the relationship between the effects of home-based PR on individuals with IPF, the associated determinants and its resultant effects on the outcomes through the conceptual framework (Fig. 1). Several determinants (involving factors related to the individual, family, and healthcare) have a direct or indirect influence on the overall well-being of the IPF individuals and the intervention (home-based PR). Considering the poor prognosis and patient-centred barriers, it is anticipated that home-based PR will bring about improvements in the functional outcomes and quality of life of individuals with IPF.

### Study methods

Electronic searches: Medline (via PubMed and Web of Science), SCOPUS, CINHAL and PEDRo will be searched for English publications until August 31, 2021. Preliminary search strategy is developed in Medline (PubMed) (Additional data), and it will be customised for other databases. Search has been reviewed by a colleague who is extensively trained by search experts from EPPI Centre and Cochrane. There will not be restriction on publication status of the articles.

Searching other resources: To identify in progress and unpublished studies ‘the WHO International Clinical Trials Registry platform’ and other country-specific trial registries, if accessible, such as ‘Clinical Trials registry of India’ will be scanned. The backward and forward citations of included studies will be scanned to look for any potentially eligible records. We will use Rayyan software for managing the data and screening the records.

Keywords: Functional capacity; Home-based pulmonary rehabilitation; Health-related quality of life; Idiopathic pulmonary fibrosis

### Data extraction and analysis

#### Study selection

Two review authors (RA and VK) will independently review titles and abstracts retrieved from the search and identify all potentially eligible studies. We will follow a piloted uniform screening protocol. Full text of included studies will be obtained, and the same team members will review independently according to the inclusion criteria. We will address disputes, if any, through discussion before consensus, and have a third review author (AM or SSP) for final decision when consensus cannot be achieved. The review authors shall document the rationale for excluding all full texts which do not meet the criteria for inclusion. The study selection process will be documented using PRISMA 2020 flow diagram (Additional data).

#### Data extraction

We will customise and use Cochrane EPOC data collection form [30] for extracting relevant information from included studies. We will follow an iterative process for piloting the data extraction form wherein all the authors will first extract the data from one included study and discuss the discrepancies in extracted data until consensus. This step will be repeated for more number of studies until authors have a thorough understanding in data extraction and are satisfied with the type of data to be extracted that would suffice for analysis. Furthermore, template for intervention description and replication (TIDieR) checklist and guide will be used to describe the intervention in detail [31]. From each included study, we will extract information on title, year of publication, author, study design, type of analysis, number of included participants, country, population (e.g. age, gender and other contextual information), severity of IPF, comorbidity, intervention (type, duration, intensity, frequency, provider etc.), comparison and outcome (follow-up, tool used, outcome assessor etc.) (Additional data). Corresponding authors of the included studies will be contacted in case of limited or lack of information in the studies. However, studies will be excluded if we do not receive a reply from the corresponding author within a fortnight. We will calculate disagreement (kappa) between reviewers for both screening and data extraction.

#### Critical appraisal of included studies

RA and VK, independently, will assess methodological quality of included studies using ‘Cochrane Risk of Bias 2’ tool [32]. Both the reviewers will clear the dissent, if any, by discussion. ROB 2 tool will assess bias at selection, blinding of participants, outcome assessment, reporting and other bias. We will judge each possible source of bias as high, low or some concern and provide a quote from the study report along with a justification. We will overcome disagreements by discussion until consensus for our decision on the ‘Risk of bias’.

#### Data synthesis

Studies will be examined for methodological, clinical and statistical heterogeneity. The statistical heterogeneity between trials will be evaluated using *I*^2^ statistics. The values will be categorised as ‘no’ (< 25%), ‘low’ (25–49%), ‘moderate’ (50–75%) or ‘substantial’ (> 75%) heterogeneity. Depending on the statistical heterogeneity, we will apply fixed or random effects model. If the statistical heterogeneity is 50 or higher, we will apply random effects model. Using RevMan, if possible, we will individually combine studies into a single meta-analysis using the generic method of inverse variance and will estimate the treatment effects reported by individual studies. Effect estimates such as odds ratios or risk ratios (for categorical data) and weighted/standardised mean differences (for continuous data) and their 95% confidence intervals will be computed. In case of substantial statistical heterogeneity or methodological and clinical heterogeneity, we will undertake narrative synthesis and subgroup analysis. Subgroups could be based on severity of IPF (or clinical variables), age, other contextual factors and study designs. Sensitivity analysis will be carried out to investigate the robustness of meta-analysis findings. We will consider variables such as risk of bias and sample size for conducting the sensitivity analysis. To assess publication bias, we will generate funnel plot and run statistical test such as Egger test [33].

We will assess certainty of evidence (high, moderate, low and very low) for each outcome measure through Grading of Recommendations Assessment, Development and Evaluation (GRADE) approach. GRADEpro GDT software will be used to generate summary of findings table [34].

## Discussion

IPF is a chronic, debilitating and fatal disease among other ILDs [1]. PR has known effects on functional capacity and quality of life in a supervised centre-based setup for individuals with IPF [9, 12, 22]. The current review will provide insights regarding the effectiveness of home-based PR on functional capacity and HRQoL in individuals with IPF.

To our best of knowledge, this would be the first systematic review to comprehensively assess the effectiveness of unsupervised home-based PR on functional capacity and quality of life in individuals with IPF. Due to restrictions in accessing the databases (e.g. Cochrane Central, PsycINFO, and other subject specific databases), search would be limited to a few databases for this systematic review. We also anticipate restriction in accessing full texts due to paywalls; however, we will contact researchers from other universities and authors of the papers to get the access. Due to resource limitations, non-English publications will not be included. We anticipate variability in terms of population and the way PR is performed in different regions, which may hinder pooling the result. To mitigate this issue, we have planned subgroup analysis. Considering the intervention defined in the review protocol, it can be anticipated that some studies might not clearly report whether the participants have been trained or the exercise training was supervised. We will contact the corresponding authors if there is limited or lack of information.

Findings from this review may help policy makers and researchers in creating supportive environment for individuals with IPF. This might activate people with IPF in home-based PR required for improving functional capacity and health-related quality of life.

## Supplementary Information

**Additional file 1.**

## Data Availability

Data sharing is not applicable to this protocol as datasets are not generated or analysed.

## Abbreviations

CRD: Chronic respiratory disease
EPOC: Effective Practice and Organisation of Care
GRADE: Grading of Recommendations Assessment, Development and Evaluation
HRQoL: Health-related quality of life
IPF: Idiopathic pulmonary fibrosis
ILD: Interstitial lung disease
PR: Pulmonary rehabilitation
PROSPERO: The International Prospective Register of Systematic Reviews
PRISMA P: Preferred Reporting Items for Systematic Review and Meta-analysis
PEDRo: The Physiotherapy Evidence Database
SF 36: Short Form 36
TIDieR: Template for Intervention Description and Replication
WHO BREF: The World Health Organisation Quality of Life Instrument
