# Supplementary material for: Effect of home-based pulmonary rehabilitation on functional capacity in people with idiopathic pulmonary fibrosis—a systematic review protocol
Source: Syst Rev. 2021 Nov 15;10:297. doi: 10.1186/s13643-021-01853-9 (PMC8591795; doi:10.1186/s13643-021-01853-9)
Supplement: Supplementary file 1 — Additional file 1. [file 13643_2021_1853_MOESM1_ESM.docx]

**Additional data**

Contents

[**1)** **Search strategy for PubMed** 1](#_Toc82427314)

[**2)** **PRISMA 2020 flow diagram** 3](#_Toc82427315)

[**3)** **Data Extraction form:** 4](#_Toc82427316)

#

# **Search strategy for PubMed**

((((((((((((((((((("Randomised controlled trial"[Title/Abstract]) OR ("Randomized controlled trial"[Title/Abstract])) OR ("cluster design"[Title/Abstract])) OR ("cluster controlled trial"[Title/Abstract])) OR ("cross-over design"[Title/Abstract])) OR ("cross over design"[Title/Abstract])) OR ("controlled clinical trial"[Title/Abstract])) OR ("quasi-randomised"[Title/Abstract])) OR ("quasi-randomized"[Title/Abstract])) OR ("quasi randomised"[Title/Abstract])) OR ("quasi randomized"[Title/Abstract])) OR ("clinical trial"[Title/Abstract])) OR ("pseudo randomised"[Title/Abstract])) OR ("pseudo-randomised"[Title/Abstract])) OR ("pseudo randomized"[Title/Abstract])) OR ("pseudo-randomized"[Title/Abstract])) OR (randomization[Title/Abstract])) OR (randomisation[Title/Abstract]) AND (english[Filter])) OR (((((clinical trial[MeSH Terms])) OR (clinical trials, randomized[MeSH Terms])) OR (controlled clinical trials, randomized[MeSH Terms])) OR (controlled clinical trial[MeSH Terms]) AND (english[Filter]) AND (english[Filter]) AND (english[Filter])) AND (english[Filter])) AND (((((((((((((((((((Exercise[MeSH Terms]) OR ("Exercise Movement Techniques"[MeSH Terms])) OR (exercise therapy[MeSH Terms])) OR (resistance training[MeSH Terms])) OR (physical education[MeSH Terms])) OR (physical education, training[MeSH Terms])) OR (self care[MeSH Terms])) OR (health education[MeSH Terms])) OR ("self-management"[MeSH Terms])) OR (resistance training[MeSH Terms])) OR ("patient-centered care"[MeSH Terms])) OR (care, self rehabilitation[MeSH Terms])) OR (center, rehabilitation[MeSH Terms]) AND (english[Filter])) OR ((((((((("home based care"[Title/Abstract]) OR ("home-based care"[Title/Abstract])) OR ("family center care"[Title/Abstract])) OR ("Patient-centered Care"[Title/Abstract])) OR ("Patient centered care"[Title/Abstract])) OR ("Patient-centric care"[Title/Abstract])) OR ("Patient centric care"[Title/Abstract])) OR ("Patient centred care"[Title/Abstract])) OR ("Patient-centred Care"[Title/Abstract]) AND (english[Filter]) AND (english[Filter]))) OR (((((rehabilitation[Title/Abstract]) OR (telerehabilitation[Title/Abstract])) OR ("tele rehabilitation"[Title/Abstract])) OR ("tele-rehabilitation"[Title/Abstract])) OR ("pulmonary rehabilitation"[Title/Abstract]) AND (english[Filter]))) OR ((((((("physical training"[Title/Abstract]) OR ("breathing exercise"[Title/Abstract])) OR ("aerobic exercise"[Title/Abstract])) OR ("resistant exercise"[Title/Abstract])) OR ("resistant training"[Title/Abstract])) OR (exercise[Title/Abstract])) OR ("physical therapy"[Title/Abstract]) AND (english[Filter]))) OR ((("self-care"[Title/Abstract]) OR ("self management"[Title/Abstract])) OR ("self-management"[Title/Abstract]) AND (english[Filter]))) OR ((("physical education"[Title/Abstract]) OR ("patient education"[Title/Abstract])) OR ("health education"[Title/Abstract]) AND (english[Filter])) AND (english[Filter])) AND ((((((((("Interstitial lung disease"[Title/Abstract]) OR ("Interstitial lung abnormality"[Title/Abstract])) OR ("Interstitial lung abnormalities"[Title/Abstract])) OR ("idiopathic pulmonary fibrosis"[Title/Abstract])) OR ("pulmonary fibrosis"[Title/Abstract])) OR ("idiopathic fibrosis"[Title/Abstract])) OR ("idiopathic interstitial pneumonia"[Title/Abstract])) OR ("lung fibrosis"[Title/Abstract]) AND (english[Filter])) OR (((("Idiopathic Pulmonary Fibrosis"[MeSH Terms]) OR ("Idiopathic Interstitial Pneumonias"[MeSH Terms])) OR ("Pulmonary Fibrosis"[MeSH Terms])) OR ("Lung Diseases, Interstitial"[MeSH Terms]) AND (english[Filter])) AND (english[Filter])) AND (english[Filter]))

# **PRISMA 2020 flow diagram**

**Identification of studies via other methods**

**Identification of studies via databases and registers**

Records identified from:

Websites (n = )

Organisations (n = )

Citation searching (n = )

etc.

Records removed *before screening*:

Duplicate records removed (n = )

Records marked as ineligible by automation tools (n = )

Records removed for other reasons (n = )

Records identified from:

Databases: Pubmed (n = ); Web of Science (n= ); SCOPUS (n=); CINHAL (n= ); PEDRo (n= )

Registers: WHO ICTRP (n= ); CTRI (n = ) etc

**Identification**

Records screened

(n = )

Records excluded

(n = )

Reports not retrieved

(n = )

Reports sought for retrieval

(n = )

Reports sought for retrieval

(n = )

Reports not retrieved

(n = )

**Screening**

Reports assessed for eligibility

(n = )

Reports excluded:

Reason 1 (n = )

Reason 2 (n = )

Reason 3 (n = )

etc.

Reports assessed for eligibility

(n = )

Reports excluded:

Reason 1 (n = )

Reason 2 (n = )

Reason 3 (n = )

etc.

Studies included in review

(n = )

Reports of included studies

(n = )

**Included**

#

# **Data Extraction form:**

| Review title or ID |  |
| --- | --- |
| Study ID |  |
| Report ID |  |

**General information:**

| Date form completed |  |
| --- | --- |
| Name/ID of person extracting data |  |
| Reference citation |  |
| Study author contact details |  |
| Publication type |  |
| Year of publication |  |
| Title |  |
| Notes: | |

**Study eligibility:**

| Study Characteristics | Eligibility criteria | | Eligibility criteria met? | | | Location in text or source |
| --- | --- | --- | --- | --- | --- | --- |
|  |  | | Yes | No | Unclear |  |
| Type of study | Randomised Controlled Trial | |  |  |  |  |
|  | 1. Parallel design | |  |  |  |  |
|  | 1. Cluster design: | |  |  |  |  |
|  | 1. Cross-over study design: | |  |  |  |  |
|  | Quasi-randomised Controlled Trial | |  |  |  |  |
|  | Before and after studies (or pre-post) with  at least 2 groups (intervention and control) | |  |  |  |  |
|  | Other design | |  |  |  |  |
| Participants | Individuals with IPF diagnosed using high resolution computed tomography findings and by registered physician.  IPF could be of any severity but, the individual should be in a stable clinical state. | |  |  |  |  |
| Types of intervention | Home-based PR (aerobic exercise, resistance exercise, or both, with or without health education)  May be performed in a group or individually in the community.  Studies with physiotherapist/any professionally trained individual periodically (minimum once in 15 days) supervising the participant, either at the participant’s home, community or hospital setup.  Web- or tele-based rehabilitation  First and single training PR session has been delivered at centre/hospital, but remaining sessions were carried out at home/community setup  Studies with individuals undergoing pharmacotherapy or any other standard care  Mixture of home-based and centre-based PR- subgroup is available for unsupervised home-based PR | |  |  |  |  |
| Types of comparison | Conventional supervised, centre-based PR treatment, no treatment or standard care  Centre-based PR, or between the providers/ supervisors.  Two different forms of home based PR (e.g., aerobic home-based PR compared to strength training home-based PR compared to strength training home-based PR) | |  |  |  |  |
| Types of outcome measures | Functional capacity using 6-minute walk distance, shuttle walk test, or incremental shuttle walk test. | |  |  |  |  |
|  | Condition specific quality of life measured using scales such as St. George respiratory questionnaire, Chronic Respiratory Distress Questionnaire and King’s Brief Interstitial Lung Disease Questionnaire or any other | |  |  |  |  |
| INCLUDE | | EXCLUDE | | | | |
| Reason for exclusion |  | | | | | |
| Notes: | | | | | | |

**Characteristics of included studies:**

**Methods:**

|  | Descriptions as stated in report/paper | | Location in text or source |
| --- | --- | --- | --- |
| Aim of study |  | |  |
| Study design |  | |  |
| Unit of allocation |  | |  |
| Start date |  | |  |
| End date |  | |  |
| Duration of participation |  | |  |
| Ethical approval needed/ obtained for study | Yes No Unclear |  |  |
| Notes: | | | |

**Participants:**

|  | Description | | Location in text or source |
| --- | --- | --- | --- |
| Population description |  | |  |
| Definition of IPF or diagnostic criteria reported in study |  | |  |
| Study Setting |  | |  |
| Inclusion criteria |  | |  |
| Exclusion criteria |  | |  |
| Method of recruitment of participants |  | |  |
| Informed consent obtained | Yes No Unclear |  |  |
| Total no. participants |  | |  |
| Clusters |  | |  |
| Baseline imbalances |  | |  |
| Withdrawals and exclusions |  | |  |
| Age |  | |  |
| Gender |  | |  |
| Severity of IPF |  | |  |
| Co-morbidities |  | |  |
| Other relevant socio-demographics |  | |  |
| Subgroup’s measure |  | |  |
| Subgroups reported |  | |  |
| Notes: | | | |

**Intervention groups:**

|  | Description as stated in report/paper | Location in text or source |
| --- | --- | --- |
| Group name |  |  |
| No. randomised to group  Or total no. of participants |  |  |
| Theoretical basis (include key references) | Is theoretical framework for designing the intervention mentioned? No  If yes, whether intervention include single theoretical framework?  Which theory is used |  |
| Description (content, dose, components) |  |  |
| Description of procedure or process used in intervention |  |  |
| Level at which intervention delivered | Group/ interpersonal |  |
| Setting/ location of delivery of intervention | (home/institution) |  |
| Duration of treatment period | Length (minutes)-  Frequency (per week)-  Duration (weeks)- |  |
| Delivery (medium or mode of delivery) | (Face-to-face/internet/telephone) |  |
| Tailoring with description (what, why, when, how) | (Personalised treatment/general regimen) |  |
| Modifications with description (what, why, when, how) | (Intervention if modified during the study course) |  |
| Assessment of intervention adherence or fidelity | How-  By whom-  Strategies used-  Extent to which intervention was delivered- |  |
| Providers (professional, their expertise) |  |  |
| Co-interventions if any (pharmacotherapy) |  |  |
| Resource requirements (equipment, educational booklet, online appendix, URL) |  |  |
| Notes: | | |

**Outcome measure:**

**Outcome 1: Functional capacity**

|  | Description as stated in report/paper | | Location in text or source |
| --- | --- | --- | --- |
| Tool used | 6-minute walk test OR  Shuttle walk test OR  Incremental shuttle walk test or specify other | |  |
| Time points measured (specify whether from start or end of intervention) |  | |  |
| Time points reported |  | |  |
| Outcome definition (with diagnostic criteria) |  | |  |
| Person measuring/ reporting |  | |  |
| Unit of measurement |  | |  |
| Scales: upper and lower limits (indicate whether high or low score is good) |  | |  |
| Is outcome/tool validated? | Yes No Unclear |  |  |
| Imputation of missing data (assumptions made for ITT analysis) |  | |  |
| Assumed risk estimate (baseline or population risk noted in Background) |  | |  |
| Power (power & sample size calculation, level of power achieved) |  | |  |
| Notes: | | | |

**Outcome 2: Quality of Life (this table will be repeated for adverse events if any)**

|  | Description as stated in the review | | Location in text or source |
| --- | --- | --- | --- |
| Tool used | St. George Respiratory Questionnaire OR  Chronic Respiratory Questionnaire OR  King’s Brief Interstitial Lung Disease Questionnaire OR specify other | |  |
| Time points measured (specify whether from start or end of intervention) |  | |  |
| Time points reported |  | |  |
| Outcome definition (with diagnostic criteria) |  | |  |
| Person measuring/ reporting |  | |  |
| Unit of measurement |  | |  |
| Scales: upper and lower limits (indicate whether high or low score is good) |  | |  |
| Is outcome/tool validated? | Yes No Unclear |  |  |
| Imputation of missing data (assumptions made for ITT analysis) |  | |  |
| Assumed risk estimate (baseline or population risk noted in Background) |  | |  |
| Power (power & sample size calculation, level of power achieved) |  | |  |
| Notes: | | | |
